# Supplementary figures and images for: Optimizing the deployment of ultra-low volume and targeted indoor residual spraying for dengue outbreak response
Source: PLoS Comput Biol. 2020 Apr 20;16(4):e1007743. doi: 10.1371/journal.pcbi.1007743 (PMC7200023; doi:10.1371/journal.pcbi.1007743)

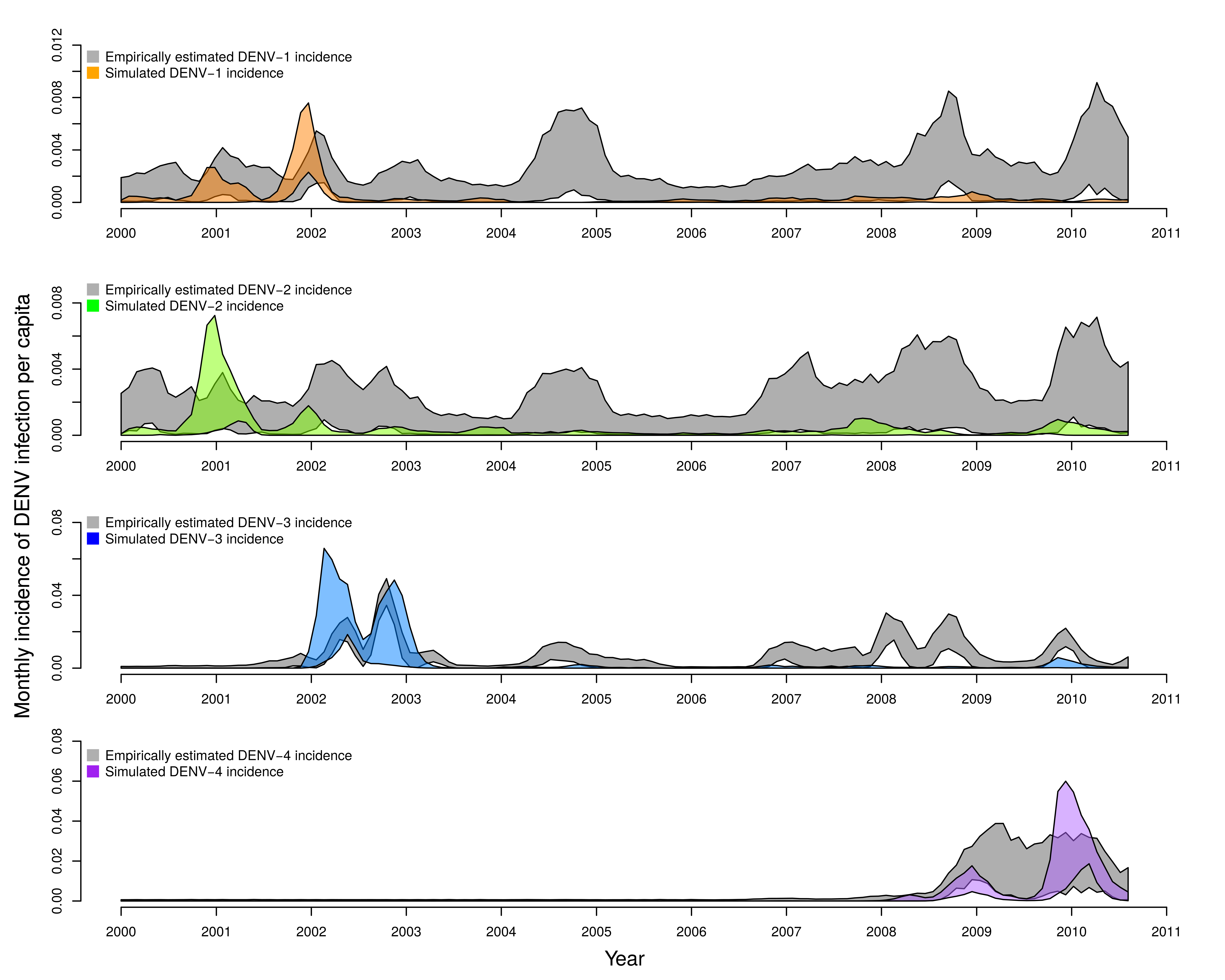

Supplement: S1 Fig — Monthly, serotype-specific incidence of infection per capita, as estimated by Reiner et al. [51](gray bands) and as reproduced by our calibrated model (colored bands). (TIF) [file pcbi.1007743.s002.tif]

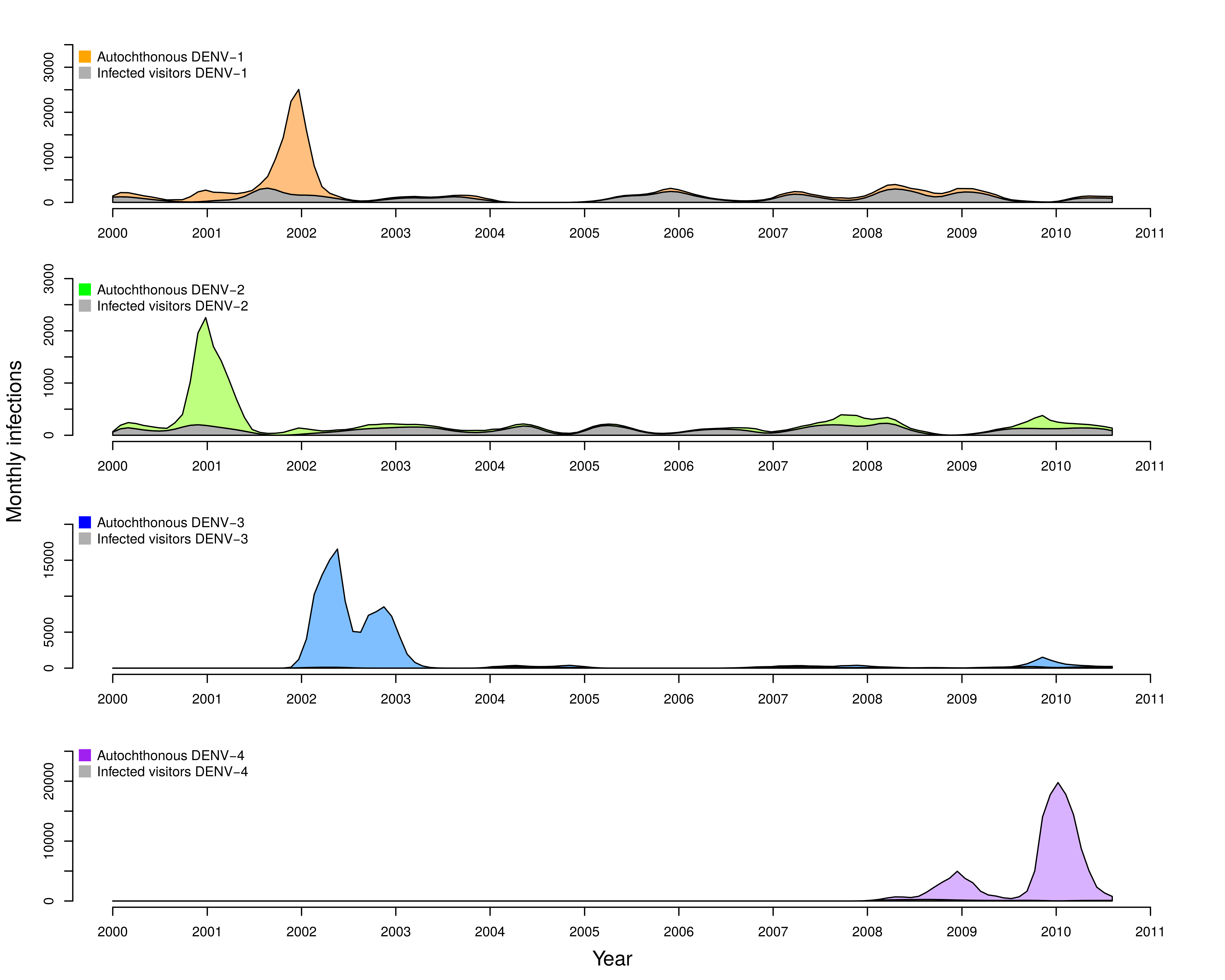

Supplement: S2 Fig — Median numbers of infections on a monthly basis for each serotype, stratified by whether the infection was acquired through biting by an infectious mosquito (colored) or by exogenously driven infections (gray) that were used to seed transmission in the model. (TIF) [file pcbi.1007743.s003.tif]

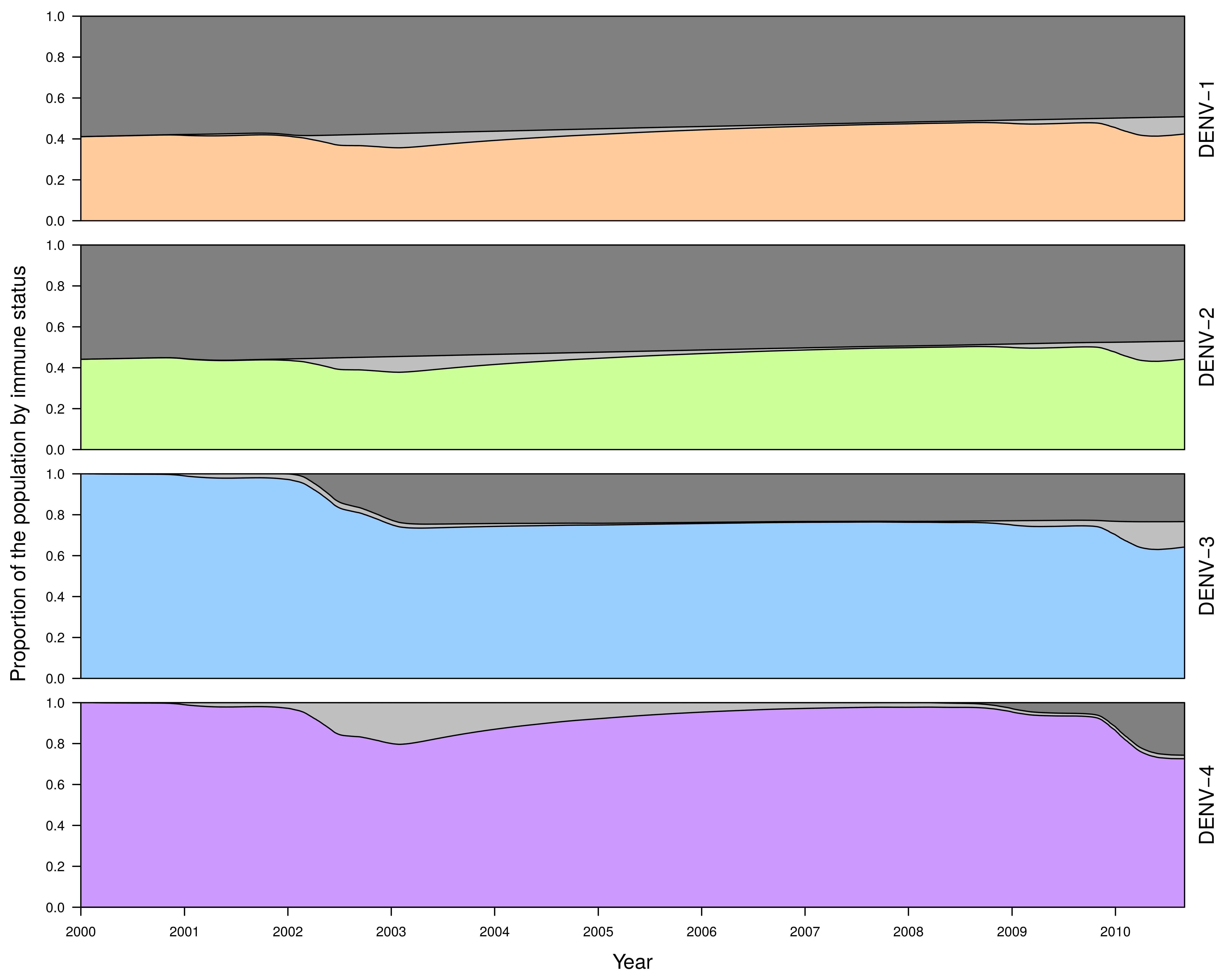

Supplement: S3 Fig — Dark gray indicates permanent homologous immunity, light gray indicates temporary heterologous immunity, and colored regions indicate susceptibility. (TIF) [file pcbi.1007743.s004.tif]

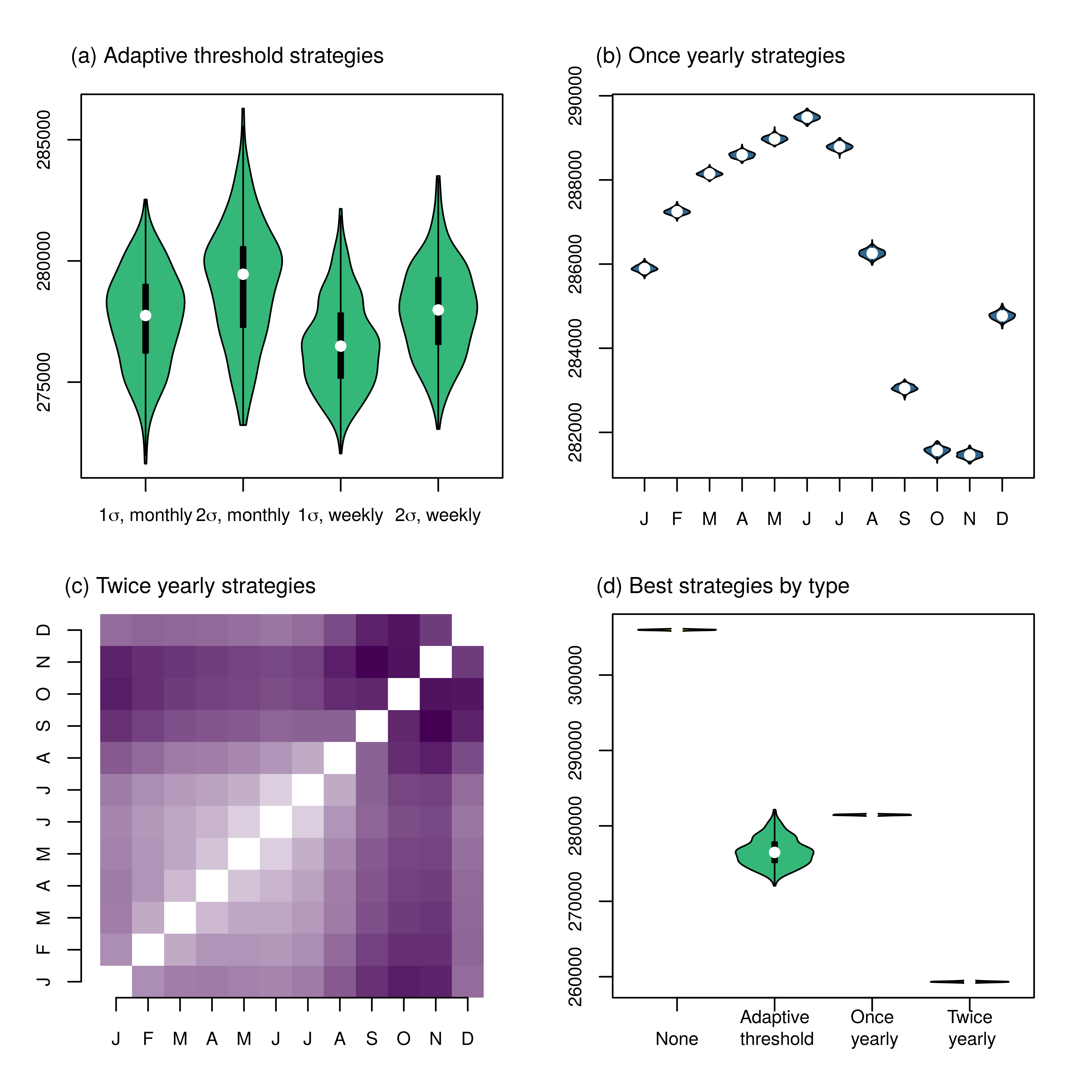

Supplement: S4 Fig — (a) Comparison of reactive strategies for initiating spraying; spraying began when the monthly or weekly incidence was one or two standard deviations above the mean for that period from the last five years, as shown on the x-axis. (b) Comparison of yearly city-wide spraying strategies, beginning on the first day of the shown month. (c) Comparison of the median predicted cases for twice yearly spraying strategies, beginning on the first days of the shown month. Darker colors correspond to fewer cases, and the diagonal shows yearly spraying strategies. (d) Comparison of the best strategies in each category: adaptive threshold corresponds to starting when monthly incidence was more than one standard deviation above the mean, once yearly corresponds to spraying in September, twice yearly corresponds to spraying in September and November. (TIF) [file pcbi.1007743.s005.tif]

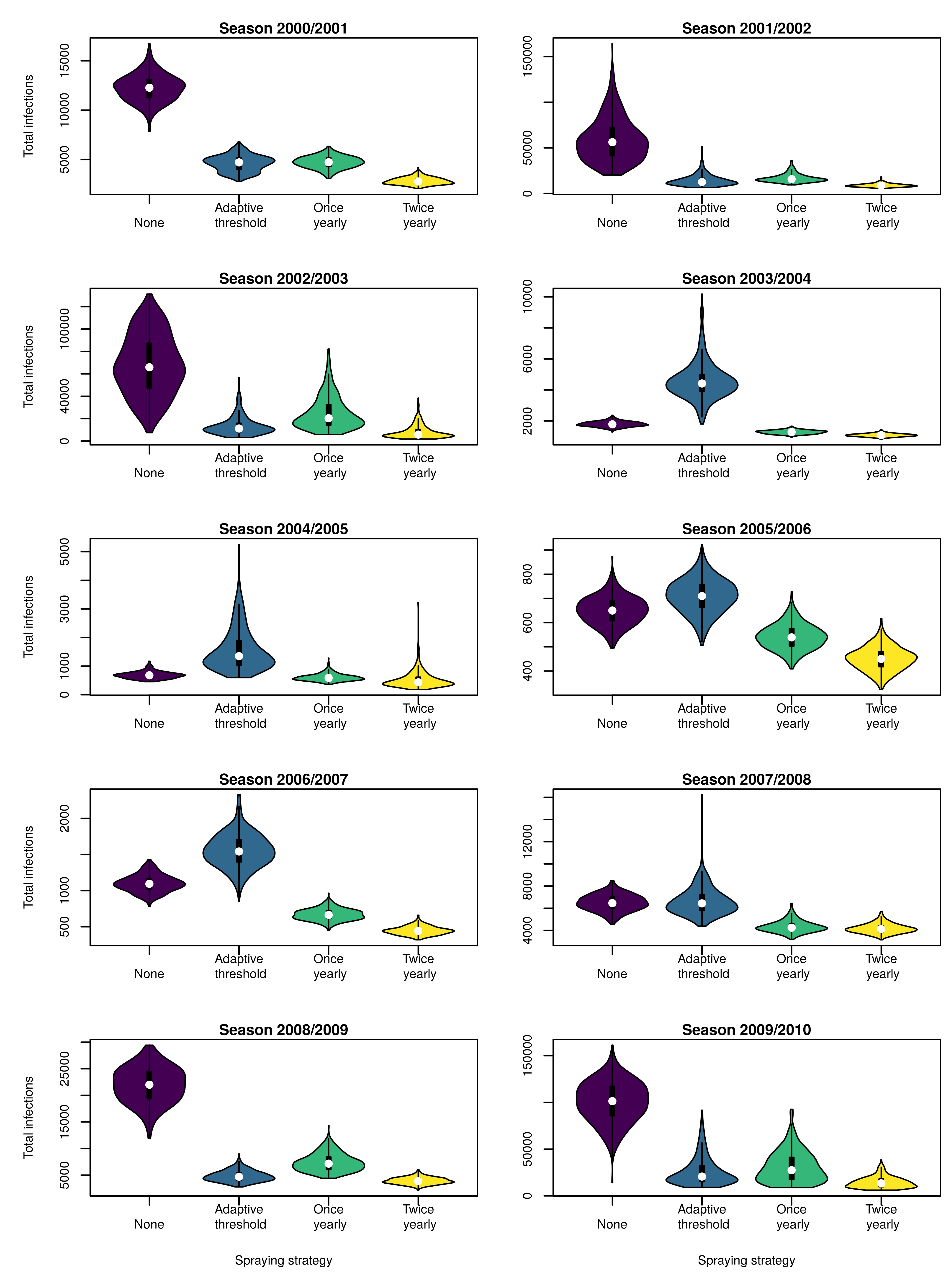

Supplement: S5 Fig — Each figure compares the best strategies in each category, for that season. (TIF) [file pcbi.1007743.s006.tif]

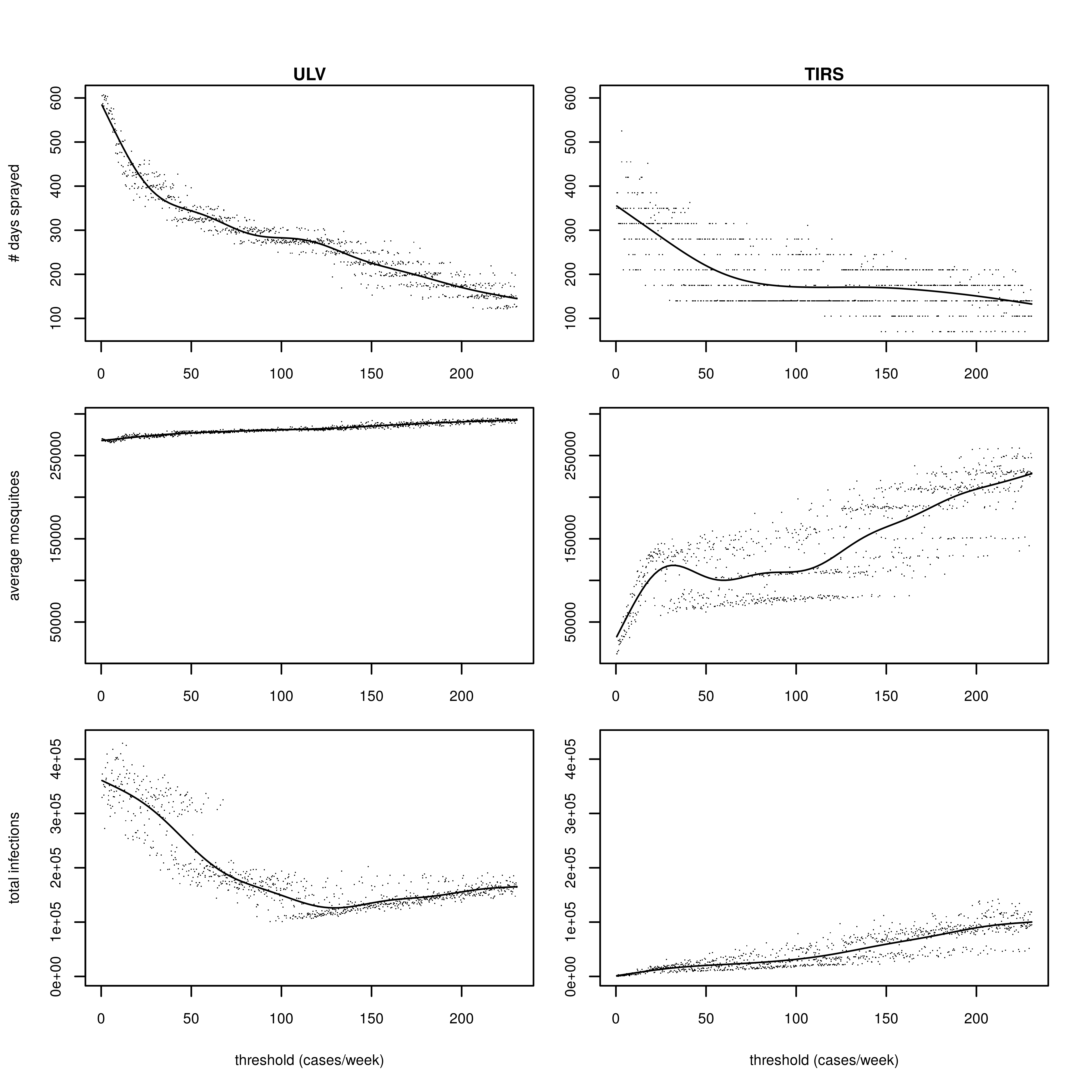

Supplement: S6 Fig — The top row shows the number of days spent spraying over the 11 year period, the middle row the mean mosquito abundance, and the bottom row the total number of dengue infections. The left column is ULV spraying and the right column TIRS. Each point represents one model simulation, and the line represents predictions by a fitted generalized additive model. (TIF) [file pcbi.1007743.s007.tif]

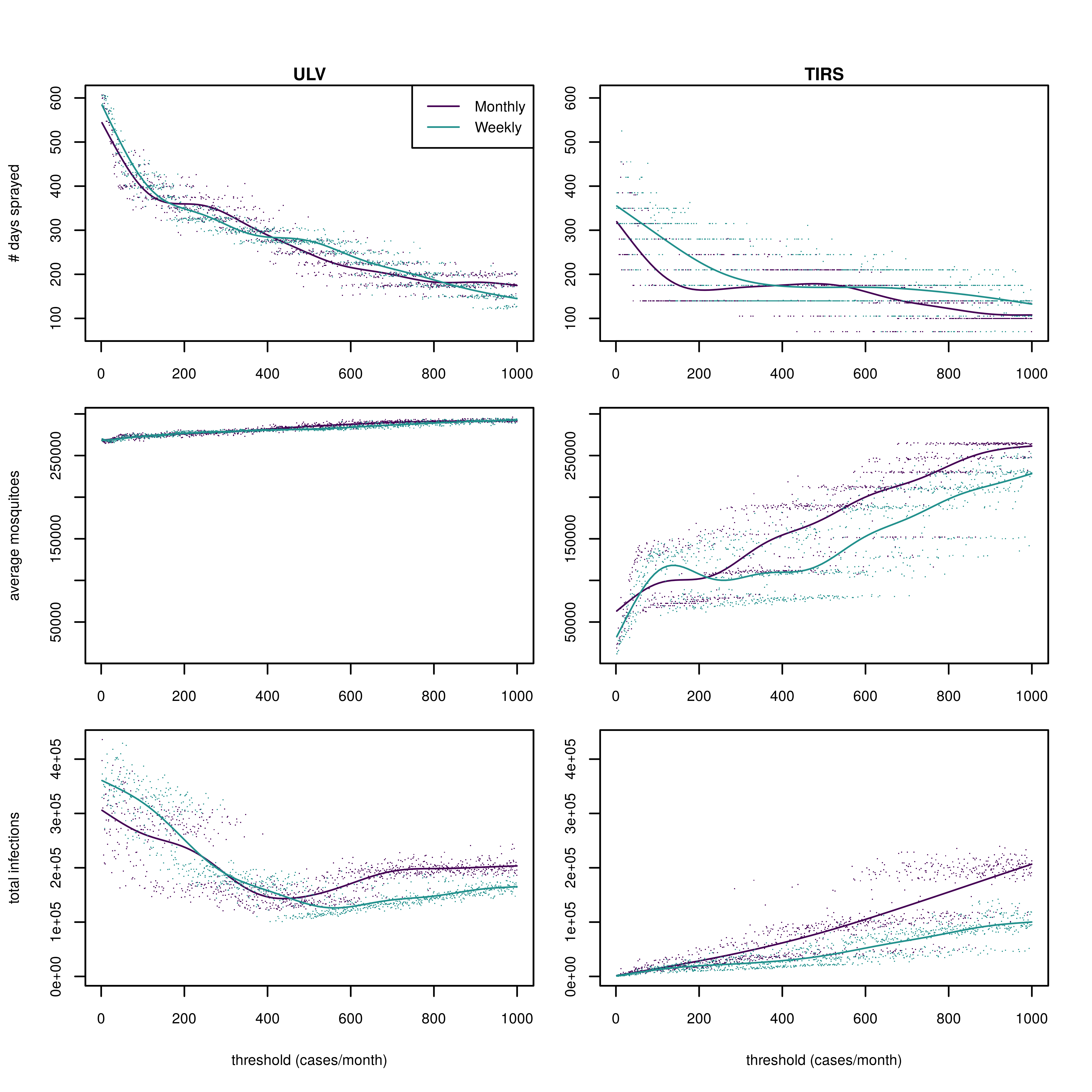

Supplement: S7 Fig — Results when initiating ULV spraying according to a fixed threshold that is monitored on a monthly (purple) or weekly (teal) basis. The top row shows the number of days spent spraying over the 11 year period, the middle row the mean mosquito abundance, and the bottom row the total number of dengue infections. The left column is ULV spraying and the right column TIRS. Each point represents one model simulation, and the line represents predictions by a fitted generalized additive model. (TIF) [file pcbi.1007743.s008.tif]

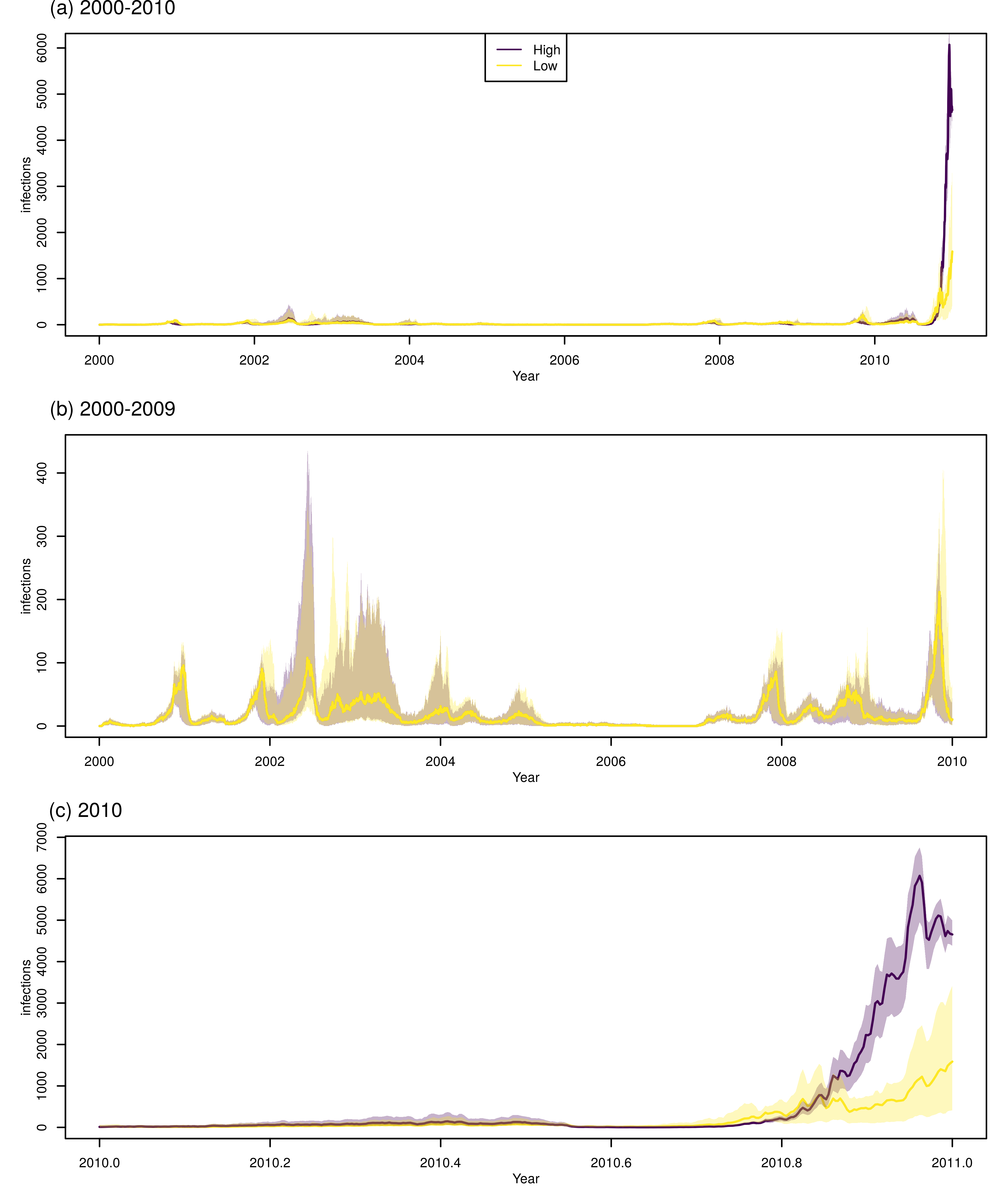

Supplement: S8 Fig — Timeseries showing median and 90% confidence intervals of simulations in different regions of the bistability observed in Fig 6 (left column). All simulations include ULV spraying with a fixed threshold between 125 and 400 cases/month. Simulations marked “high” (purple) are those for which the total number of infections was above 210,000, and “low” are those below this value. Panels (b) and (c) show detail of panel (a). (TIF) [file pcbi.1007743.s009.tif]

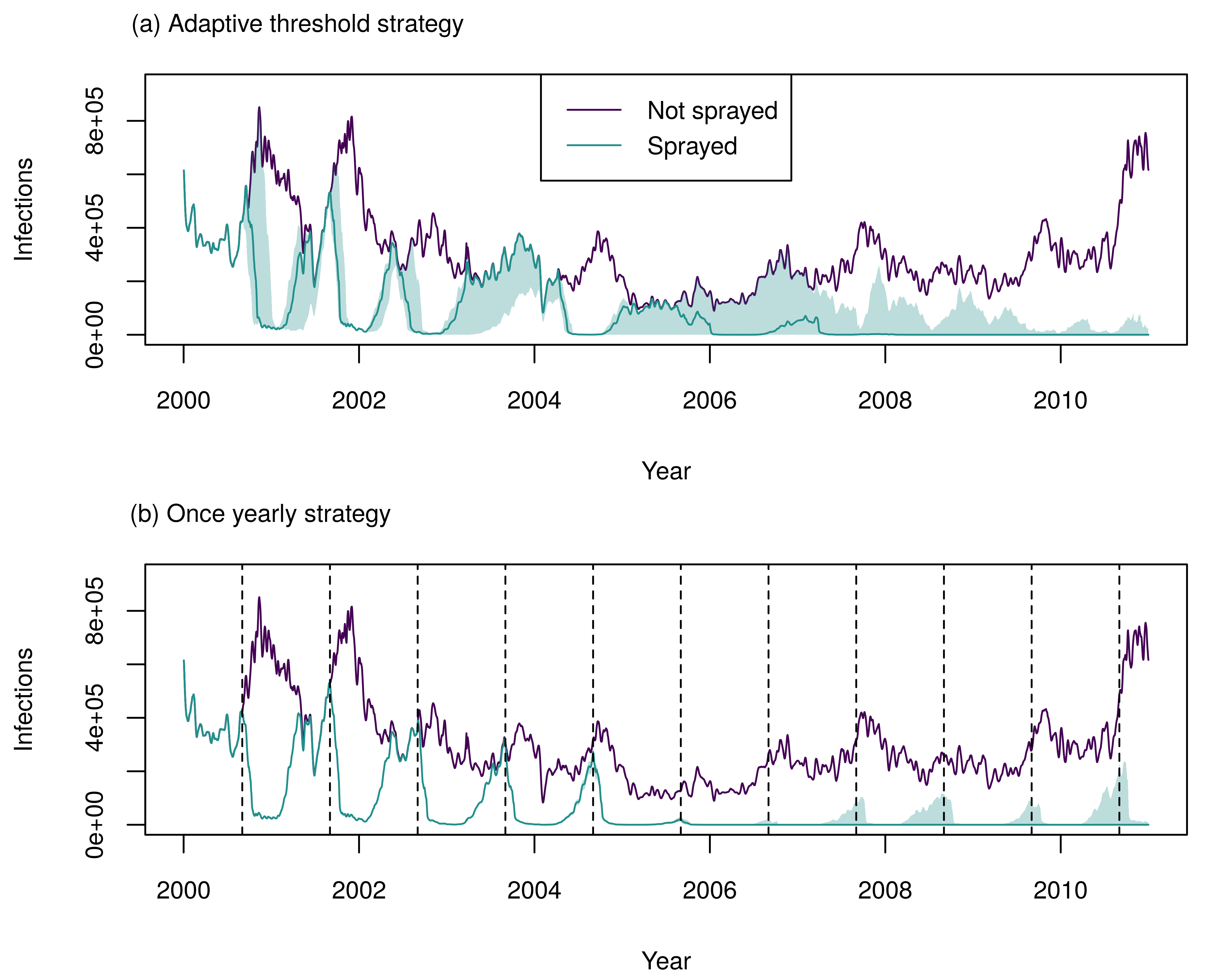

Supplement: S9 Fig — Time-series of mosquito abundance for the best TIRS strategy for (a) the best adaptive threshold strategy (starting when weekly incidence exceeds the mean by 1σ) and (b) the best yearly strategy (starting in September). In each plot the purple lines represent the predictions without spraying, and the green represents the given strategy. The line represents the median of all 400 simulations and the shading represents the inter-quartile range. (TIF) [file pcbi.1007743.s010.tif]

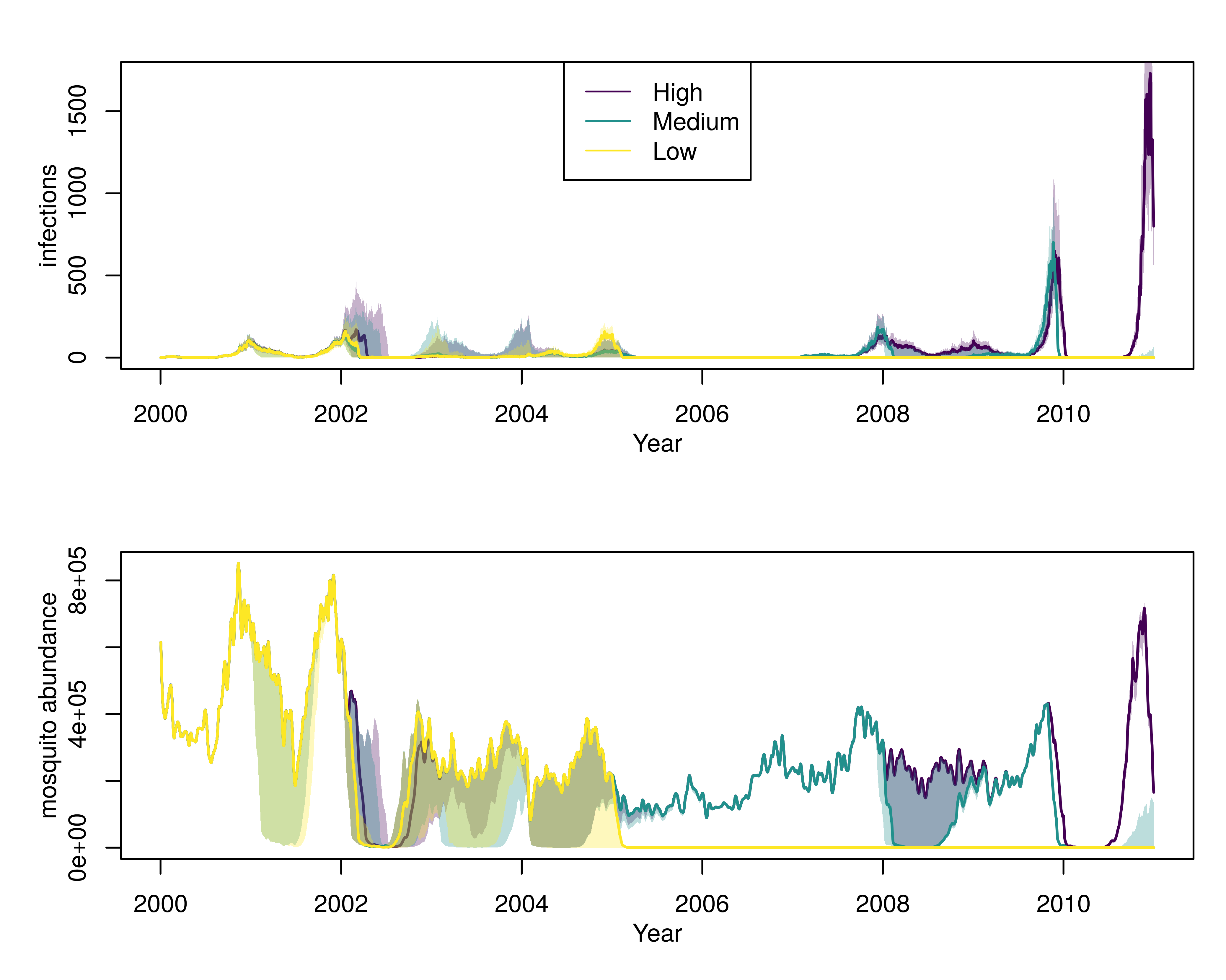

Supplement: S10 Fig — All simulations include TIRS spraying with a fixed threshold above 500 cases/month. Simulations marked “high” (purple) are those for which the total number of infections was above 150,000, “medium” (teal) are those between 75,000 and 150,000 and “low” (yellow) are those below 75,000. The top panel shows number of infections and the bottom panel mosquito abundance. (TIF) [file pcbi.1007743.s011.tif]

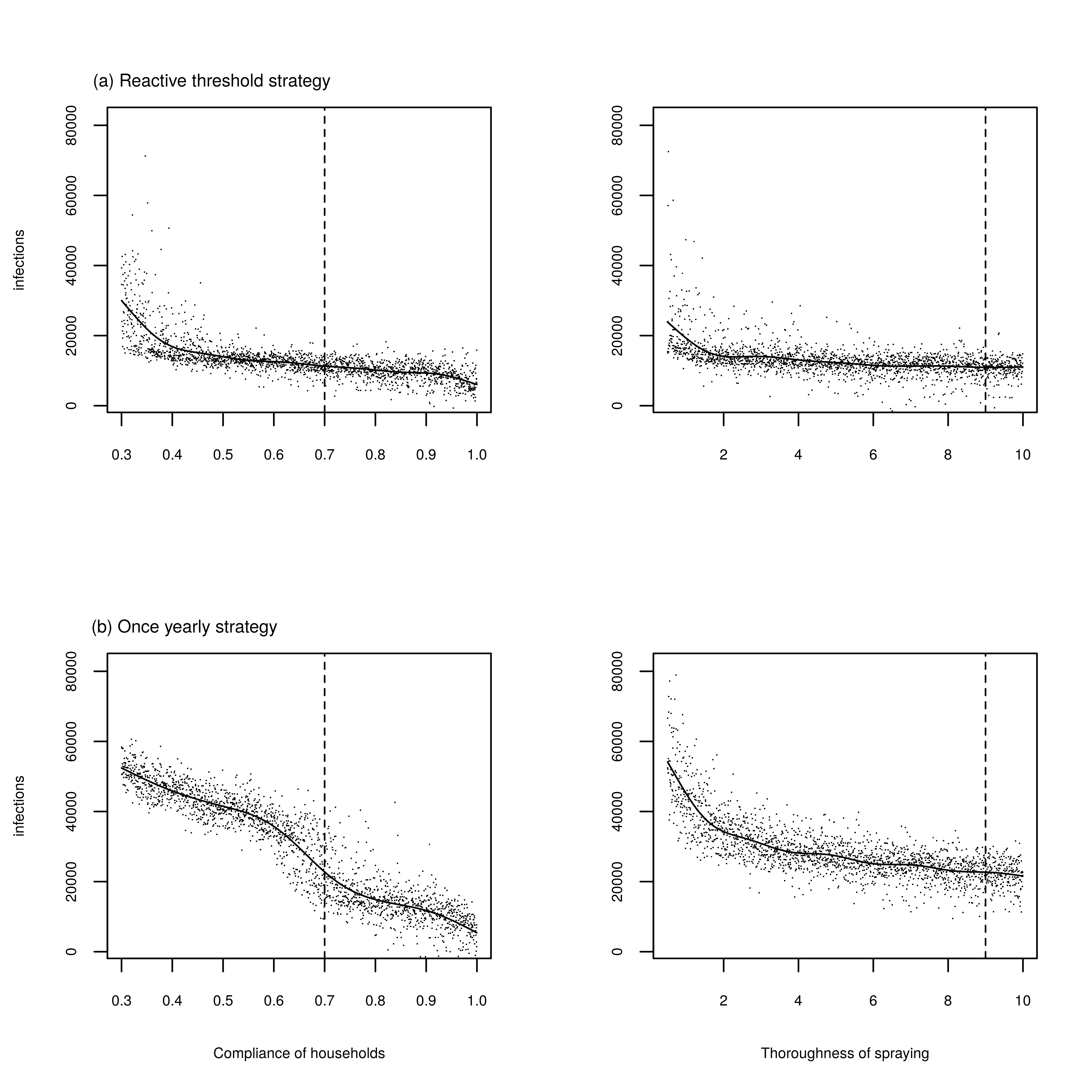

Supplement: S11 Fig — The vertical line shows the value used in the baseline simulations. The solid line represents a fitted multivariable generalized additive model. (TIF) [file pcbi.1007743.s012.tif]

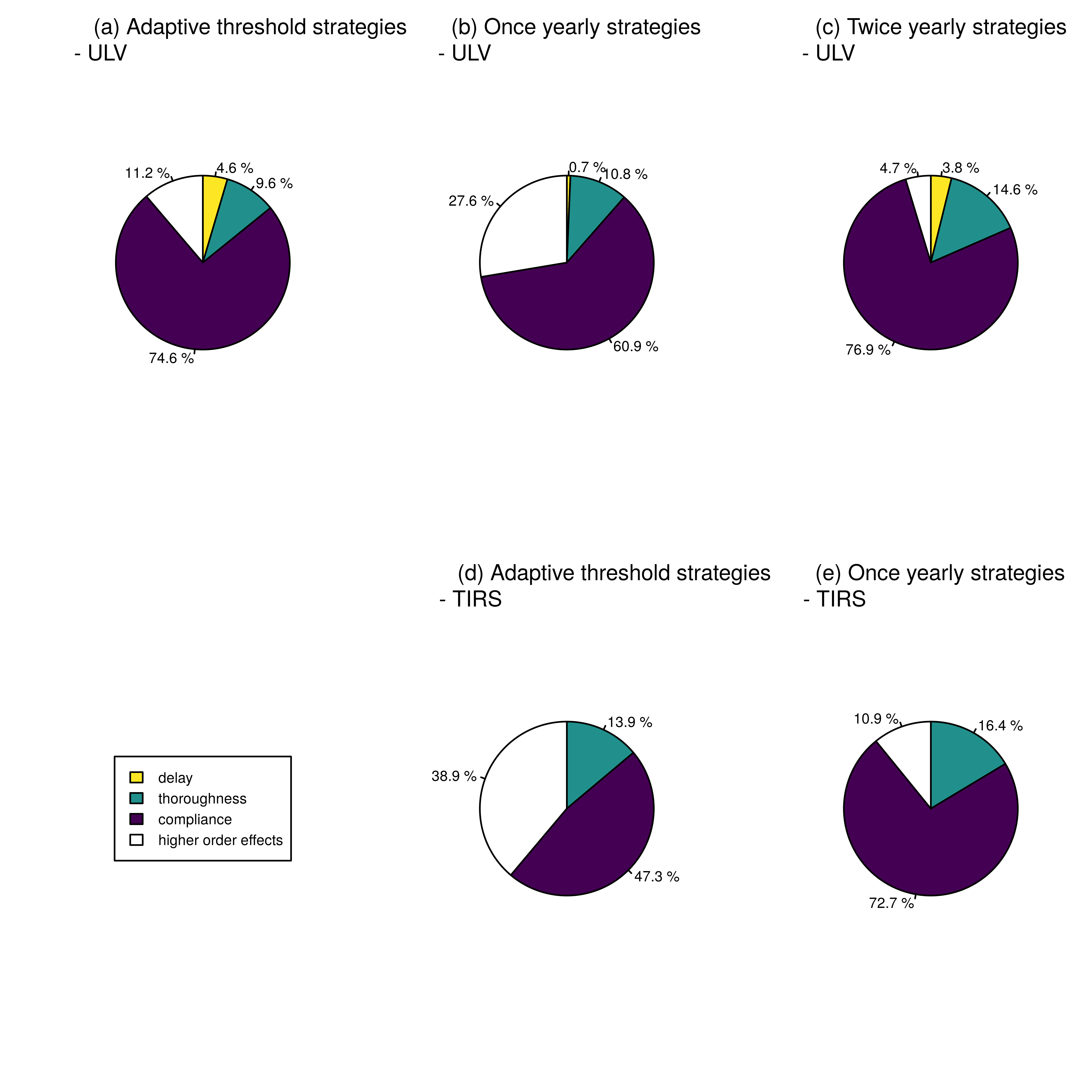

Supplement: S12 Fig — Pie charts showing the proportion of variance in the output that is explained by variance in the sampled input parameters for (a) adaptive threshold ULV strategies, (b) yearly ULV strategies, (c) twice yearly ULV strategies, (d) adaptive threshold TIRS strategies, and (e) yearly TIRS strategies. Higher-order terms include interactions between parameters as well as aleatory uncertainty; in the case of TIRS the only interaction is the interaction between compliance and thoroughness as only two parameters were varied. (TIF) [file pcbi.1007743.s013.tif]
