# Supplementary material for: Optimizing the deployment of ultra-low volume and targeted indoor residual spraying for dengue outbreak response
Source: PLoS Comput Biol. 2020 Apr 20;16(4):e1007743. doi: 10.1371/journal.pcbi.1007743 (PMC7200023; doi:10.1371/journal.pcbi.1007743)
Supplement: Supplementary file 3 [file pcbi.1007743.s001.odt]

# S1 Text: Further details of the agent-based model of dengue virus transmission.

An ABM consists of “agents” (in this case, people and mosquitoes) who interact with each other in a shared environment. The environment of our model was represented by climate conditions and a set of locations, such as houses, schools, parks, cemeteries, and churches. Climate conditions affected mosquito biting frequency, survival, and incubation time of DENV. Locations represented all 92,891 buildings in the city. For about 38,835 of these locations, mainly in the central portion of the city, we had data on exact coordinates and location type. For the remainder, we randomly distributed the locations and randomly assigned a location type. Agents represented approximately 450,000 individuals that live in this area [1].

Our synthetic population realistically portrayed the population of Iquitos in terms of demographic characteristics of how people are distributed across houses and over time. Specifically, the demographic profiles of the modeled households were consistent with survey data collected during a previous study [2]. The population-wide sex and age distributions were consistent with U.N. estimates for Peru. To represent population changes in time, we simulated human births and deaths that match those estimated by the U.N. for Iquitos, while simultaneously preserving realistic household compositions by placing newborn children in houses with appropriately aged mothers as determined by U.N. estimates of age-specific fertility of Peru [1]. For each person, we simulated daily human movement patterns with a model previously described by Perkins et al. [3], which was fitted to data from retrospective, semi-structured interviews of residents of Iquitos [4,5].

We modeled three immature mosquito stages (eggs, larvae, and pupae) deterministically at each location. Development and mortality rates for each of these stages, along with adult mortality rates and the rate at which pupae emerged as adults, varied daily, and are based on empirically derived temperature-dependent relationships and weather data from Iquitos [6,7]. Additionally, larval density dependent mortality and an additional rate of death in both the larval and pupal stages are calibrated so that adult mosquito abundance matches the spatio-temporal heterogeneity predicted by a previous study from Iquitos [8]. Adult mosquitoes blood-fed on humans who were co-located with a mosquito during its time of blood-feeding at rates informed by empirical relationships with temperature [7,9]. Each day, mosquitoes moved to adjacent locations based on a probability of 0.3, consistent with another agent-based model of Ae. aegypti population dynamics in Iquitos [6]. 

The transmission of DENV to humans, and to mosquitoes, occurred through mosquito bites. The probability of transmission from humans to mosquitoes was determined by the viremia levels of the infecting human at the time of the bite [10]. After completion of a temperature-dependent extrinsic incubation period [11], infectious mosquitoes transmitted DENV to susceptible humans with a fixed probability of 1.0 [12]. Infected humans became infectious and, with a probability informed by empirical studies [13], developed symptoms following a latency period linked to the timing of peak viremia [11]. After recovering from infection, humans gained permanent immunity to the infecting serotype and temporary immunity against heterotypic infections for a period of time. The duration of temporary immunity was exponentially distributed across people with a mean of 686 days, as estimated by a previous model-based analyses of serotype-specific dengue incidence time series [14]. We assigned the initial level of population immunity to each serotype in the population based on estimates by Reiner et al. [15].

References:

1. 	Perkins TA, Reiner RC, España G, Ten Bosch QA, Verma A, Liebman KA, et al. An agent-based model of dengue virus transmission shows how uncertainty about breakthrough infections influences vaccination impact projections. PLoS Comput Biol. 2019;15: e1006710. doi:10.1371/journal.pcbi.1006710

2. 	Liebman KA, Stoddard ST, Reiner RC, Perkins TA, Astete H, Sihuincha M, et al. Determinants of Heterogeneous Blood Feeding Patterns by Aedes aegypti in Iquitos, Peru. Barrera R, editor. PLoS Neglected Tropical Diseases. 2014;8: e2702. doi:10.1371/journal.pntd.0002702

3. 	Perkins TA, Garcia AJ, Paz-Soldán VA, Stoddard ST, Reiner RC, Vazquez-Prokopec G, et al. Theory and data for simulating fine-scale human movement in an urban environment. J R Soc Interface. 2014;11. doi:10.1098/rsif.2014.0642

4. 	Paz-Soldan VA, Stoddard ST, Vazquez-Prokopec G, Morrison AC, Elder JP, Kitron U, et al. Assessing and maximizing the acceptability of global positioning system device use for studying the role of human movement in dengue virus transmission in Iquitos, Peru. Am J Trop Med Hyg. 2010;82: 723–730. doi:10.4269/ajtmh.2010.09-0496

5. 	Paz-Soldan VA, Reiner RC, Morrison AC, Stoddard ST, Kitron U, Scott TW, et al. Strengths and weaknesses of Global Positioning System (GPS) data-loggers and semi-structured interviews for capturing fine-scale human mobility: findings from Iquitos, Peru. PLoS Negl Trop Dis. 2014;8: e2888. doi:10.1371/journal.pntd.0002888

6. 	Magori K, Legros M, Puente ME, Focks DA, Scott TW, Lloyd AL, et al. Skeeter Buster: a stochastic, spatially explicit modeling tool for studying Aedes aegypti population replacement and population suppression strategies. PLoS Negl Trop Dis. 2009;3: e508. doi:10.1371/journal.pntd.0000508

7. 	Otero M, Solari HG, Schweigmann N. A stochastic population dynamics model for Aedes aegypti: formulation and application to a city with temperate climate. Bull Math Biol. 2006;68: 1945–1974. doi:10.1007/s11538-006-9067-y

8. 	Reiner RC, Stoddard ST, Vazquez-Prokopec GM, Astete H, Perkins TA, Sihuincha M, et al. Estimating the impact of city-wide Aedes aegypti population control: An observational study in Iquitos, Peru. PLoS Negl Trop Dis. 2019;13: e0007255. doi:10.1371/journal.pntd.0007255

9. 	Focks DA, Haile DG, Daniels E, Mount GA. Dynamic Life Table Model for Aedes aegypti (Diptera: Culicidae): Analysis of the Literature and Model Development. J Med Entomol. 1993;30: 1003–1017. doi:10.1093/jmedent/30.6.1003

10. 	Ten Bosch QA, Clapham HE, Lambrechts L, Duong V, Buchy P, Althouse BM, et al. Contributions from the silent majority dominate dengue virus transmission. PLoS Pathog. 2018;14: e1006965. doi:10.1371/journal.ppat.1006965

11. 	Chan M, Johansson MA. The incubation periods of Dengue viruses. PLoS ONE. 2012;7: e50972. doi:10.1371/journal.pone.0050972

12. 	Ellis AM, Garcia AJ, Focks DA, Morrison AC, Scott TW. Parameterization and Sensitivity Analysis of a Complex Simulation Model for Mosquito Population Dynamics, Dengue Transmission, and Their Control. Am J Trop Med Hyg. 2011;85: 257–264. doi:10.4269/ajtmh.2011.10-0516

13. 	Clapham HE, Cummings DAT, Johansson MA. Immune status alters the probability of apparent illness due to dengue virus infection: Evidence from a pooled analysis across multiple cohort and cluster studies. PLoS Negl Trop Dis. 2017;11: e0005926. doi:10.1371/journal.pntd.0005926

14. 	Reich Nicholas G., Shrestha Sourya, King Aaron A., Rohani Pejman, Lessler Justin, Kalayanarooj Siripen, et al. Interactions between serotypes of dengue highlight epidemiological impact of cross-immunity. Journal of The Royal Society Interface. 2013;10: 20130414. doi:10.1098/rsif.2013.0414

15. 	Reiner RC, Stoddard ST, Forshey BM, King AA, Ellis AM, Lloyd AL, et al. Time-varying, serotype-specific force of infection of dengue virus. Proc Natl Acad Sci USA. 2014;111: E2694-2702. doi:10.1073/pnas.1314933111
