# Supplementary material for: Optimizing the deployment of ultra-low volume and targeted indoor residual spraying for dengue outbreak response
Source: PLoS Comput Biol. 2020 Apr 20;16(4):e1007743. doi: 10.1371/journal.pcbi.1007743 (PMC7200023; doi:10.1371/journal.pcbi.1007743)
Supplement: Attachment — Submitted filename: PCOMPBIOL-D-19-01721.pdf [file pcbi.1007743.s014.pdf]

Optimizing the deployment of ultra-low volume and indoor residual spraying for dengue outbreak response.

This is an interesting paper, which adds to a series of papers focusing on dengue transmission in Iquitos, Perú. In general, the paper is well written. It could be improved, however, to facilitate reading and to better focus its message in the different sections.

See below suggestions and particular examples that could help improve the paper.

1. More details about Iquitos are warranted. In particular for this work, it is fundamental to know if during the explored period some type of campaign against dengue was occurring in the city (e.g. spraying or control of *Aedes aegypti* breeding sites). Because the underlying transmission model is calibrated from data, if a campaign was occurring the results would reflect the combination/interplay of this campaign and the numerical "experiments".

2. The Method section is a bit confusing. Please consider the following examples of how it could be improved.
   2.1. The addition of a diagram illustrating/summarizing the model in general (scales? inputs of the model? e.g. real temperature, rainfall?), including in more detail the spraying modeling introduced in this paper. The authors already give references to other publications, but it would be useful for the reader to have a general diagram here. Moreover, from the references it is difficult to obtain a sufficiently clear picture of the underlying epidemiological model used here without extensive reading.
   2.2. It would be useful to explicitly mention some periods of time that arise in the text. E.g:
   - line 141: which data (mosquito, incidence?) and period have been used for the calibration? the same period used for the analysis (2000-2010)? Calibration of what particular parameters? which ones or at least related to what. Could you show a plot of the calibration (in supporting information)?
   - line 117: "After a period of time, another cycle…" please detail this period of time.
   - line 178: What number of cycles? For example, x number per year/month/week, etc.
   - line 184: "..., based on records of past city-wide spraying campaigns, …" Reference? period?

   2.3. Sensitive analysis:
   - Line 240, are the scenarios of 10% reporting and a lag of two weeks for notification the same ones used for calibration? Is it possible to give a reference for these assumptions?

   2.4. Spraying parameters:
   - They are not sufficiently clear before line 232. Their values are not clearly reported.

- I suggest to include the parameters and their values (and/or explored ranges) in a table (with some references?). These could be included in table 1.
- In line 233 the sentence “...plausible values…” is too vague.

3. Results.
   3.1. I would appreciate a comparison with the real reported number of infections, for example this could be added on table 2. Also, a real time series of dengue incidence in Iquitos would be valuable, which could be included in Figure 4.
   3.2. Some axes names in Figure 3, Figure 4, and Figure 7 are missing.
   3.3. It might be a good idea to combine Figure 3 and Figure 7 to facilitate comparison between methods. In fact, in line 333, the authors ask to compare these two figures “…,(compare Figure 7 to Figure 3).”
   3.4. The right and left columns of Figures 5 and 9 could be condensed into one. That is, the monthly and weekly results could be plotted in one graph, which would facilitate the comparison. Moreover, it may be good to produce only one figure with the information of figure 5 in one column and information of figure 9 in another column.
   3.5. The second row, the mosquito abundance, in Figure 5 is not discussed. In particular, I would appreciate a discussion on why as the threshold increases, the abundance of mosquitoes increases while the total number of infections “decreases” (I understood that it has a minimum for middle values). Moreover, with the TIRS (figure 9) this is different, both mosquitoes abundances and total infections increases as the threshold is higher. In addition, Figure 9 is practically not discussed in the results section (only one sentence).

4. Discussion. The discussion is broad, but it is a somewhat long, it could also be better organized. I suggest working on the writing of this section so that the main ideas are more straightforward.
