# Supplementary material for: Optimizing the deployment of ultra-low volume and targeted indoor residual spraying for dengue outbreak response
Source: PLoS Comput Biol. 2020 Apr 20;16(4):e1007743. doi: 10.1371/journal.pcbi.1007743 (PMC7200023; doi:10.1371/journal.pcbi.1007743)
Supplement: Attachment — Submitted filename: outbreak_response_reviewers_comments_20200130.docx [file pcbi.1007743.s015.docx]

# Response to reviewers

In the below, ordinary text denotes reviewer comments, **bold text our responses**, and red text passages added to the main document. Line numbers refer to the corrected version, not the tracked changes version.

Reviewer #1: Summary
Application of ultra-low volume spraying remains the primary intervention strategy against dengue. The authors demonstrate that targeted indoor residual spraying can more effectively control dengue using a mathematical model calibrated to data from Iquitos, Peru.

I thoroughly enjoyed reading this paper and would recommend it for publication given the following (very) minor revisions aimed at clarifying figures and explaining model dynamics.

**We thank the reviewer for their kind words and helpful recommendations. Please see below for responses to these comments**

Minor points:
1. The first citation in the paper on line 64 is inappropriate for the statement. The cited paper only presents several outbreaks in one Brazilian. Please can the authors consider a different reference giving an idea of rising global dengue incidence.

**We added references to articles by Stanway et al. and Brady & Hay that reviews the expansion and rise of dengue.**

2. Although most axis labels in Figures 3, 4 and 7 are the same, please add axis labels.

**Axis labels are now included.**

3. For Figure 3c, please can the authors consider adding a colourbar legend so the reader can more easily compare total predicted human infections with other subfigures.

**A colorbar is now included.**

4. The bi-stable behavior of the total number of infections at low thresholds (around 200 cases per month) with ULV spraying (Figure 5, bottom panels) is interesting. Is this due to the stochasticity of the model? I.e. in some simulations is the quota used up before a large outbreak, but in other simulations is there just enough sprays remaining to avert these infections?

**This is an interesting observation. We looked separately at the simulations in the upper and lower regions of the bistability. It seems that the higher infection numbers occur in those simulations in which spraying fails to mitigate the large epidemic in 2010, whereas in the lower region, this epidemic is largely mitigated. We added a supplementary figure and the following text to discuss this observation:**

“At low thresholds, there was bistability in the total number of infections. We explored this by looking separately at those simulations for which the total number of infections was above 210,000, and those for which it was below this value (Supplementary Figure 8). In both cases, we limited our analyses to simulations for which the threshold was between 125 and 400, the region in which the bistability occurs. This additional analysis indicated that the bistability was due to the stochastic nature of the model, and in particular, the extent to which the large epidemic in 2010 is mitigated.” (lines 342-348)

5. Similarly, can the authors explain the tri-stable dynamics of the total number of infections at large thresholds (600 cases per month and above) with TIRS.

**We explored this tristability in a similar way to the ULV bistability. In this case, it seems that the difference between the intermediate and upper regions of the tristability is again due to the extent to which the 2010 epidemic is mitigated. In this case, the lowest region corresponds to those simulations in which *Aedes aegypti* are actually eliminated from Iquitos in 2005, and we see no further transmission after then. We added supplementary figures and the following text to explain this.**

“For thresholds above 500 cases per month, there was a tristability in the total number of infections. To explore this, we stratified the simulations that had a threshold above 500 into three groups based on the number of infections (Supplementary Figure 10). Similar to the bistability observed for ULV spraying, the difference between the simulations with intermediate number of infections and those with high number of infections was due to whether the large epidemic in 2010 was averted or not. Simulations with low total number of infections were due to total elimination of *Ae. aegypti* from Iquitos in 2005 (Supplementary Figure 10). Hence, in those simulations, we observed no further dengue virus transmission following the elimination.” (lines 380-388)

Reviewer #2:

This is an interesting paper, which adds to a series of papers focusing on dengue transmission in Iquitos, Perú. In general, the paper is well written. It could be improved, however, to facilitate reading and to better focus its message in the different sections.

**The reviewer makes a number of useful suggestions, for which we are grateful. See below for detailed responses to these comments.**

See below suggestions and particular examples that could help improve the paper.

1. More details about Iquitos are warranted. In particular for this work, it is fundamental to know if during the explored period some type of campaign against dengue was occurring in the city (e.g. spraying or control of Aedes aegypti breeding sites). Because the underlying transmission model is calibrated from data, if a campaign was occurring the results would reflect the combination/interplay of this campaign and the numerical “experiments”.

**During the period of interest, household spraying efforts were ongoing in Iquitos. We added the following text to the Methods to clarify this.**

“During this period, household spraying efforts were ongoing in Iquitos [52]. The spatio-temporal pattern in mosquito abundance was calibrated to a counterfactual surface, which removed the effect of this spraying, though it was present in the particle filter calibration of the number of imported cases.” (lines 151-154)

**We also added the following comments to the Discussion to describe the limitation you point out regarding the fact that the model is partially calibrated to data which includes spraying.**

“A third limitation is that the force of importation and magnitude of mosquito abundance is calibrated to an estimate of the force of infection, which includes spraying events that took place in 2000-2010. This may mean that we overestimated the effect of spraying, as our baseline in effect already includes spraying.” (lines 555-558)

2. The Method section is a bit confusing. Please consider the following examples of how it could be improved.

2.1. The addition of a diagram illustrating/summarizing the model in general (scales? inputs of the model? e.g. real temperature, rainfall?), including in more detail the spraying modeling introduced in this paper. The authors already give references to other publications, but it would be useful for the reader to have a general diagram here. Moreover, from the references it is difficult to obtain a sufficiently clear picture of the underlying epidemiological model used here without extensive reading.

**We added a figure (Figure 1) to provide an overview of both the model and the spraying protocol.**

2.2. It would be useful to explicitly mention some periods of time that arise in the text. E.g:

- line 141: which data (mosquito, incidence?) and period have been used for the calibration? the same period used for the analysis (2000-2010)?Calibration of what particular parameters? which ones or at least related to what. Could you show a plot of the calibration (in supporting information)?

**We have added the following lines of text to clarify this. We also added three supplementary figures to show the results of the main calibration (Supplementary Figures 1-3).**

“We calibrated the initial level of population immunity, number of imported infections, and the absolute mosquito abundance using a particle filtering approach to an estimate of the serotype-specific number of infections based on a longitudinal cohort study in Iquitos [51]. Results of this calibration procedure are shown in Supplementary Figures 1-3, including a comparison of model predicted numbers of infections and the estimated number to which we calibrated in Supplementary Figure 1. Full details of this calibration procedure are given in Perkins et al. [49]. Several other components of the model were independently calibrated to data from Iquitos, including the spatio-temporal pattern in mosquito abundance [52] and human movement [53].” (lines 142-150)

- line 177: “After a period of time, another cycle...” please detail this period of time.

**The period of time between cycles was varied, but in the baseline scenario it was zero. We explore leaving a gap between cycles in the sensitivity analysis. The text has been modified in the following ways to clarify this:**

“Immediately after all zones are sprayed, another cycle of spraying the 35 zones in batches is initiated...” (lines 195-196)

“We explore the effect of leaving a gap between spray cycles in the sensitivity analysis.” (lines 179-197-198)

- line 178: What number of cycles? For example, x number per year/month/week, etc.

**We amended the text in the following way to clarify this:**

“… until a fixed number of cycles have been completed (three cycles for ULV, or one cycle for TIRS).” (lines 196-197)

- line 184: “..., based on records of past city-wide spraying campaigns, ...” Reference? Period?

**These refer to internal naval records and are not publicly available. We slightly amended the text** **to clarify this:**

**“**The most realistic parameterization, based on internal US Naval records of past city-wide spraying campaigns that took place in 2014, involved attempting to spray 11,000 houses per day on average, of which ~6,800 (62%) were compliant.” (lines 203-206)

2.3. Sensitive analysis:

- Line 240, are the scenarios of 10% reporting and a lag of two weeks for notification the same ones used for calibration? Is it possible to give a reference for these assumptions?

**These values only affect the calculation of the adaptive threshold, and whether it is passed, and so do not affect the calibration. Additionally, the model was calibrated to serotype-specific estimates of infections that were based on serology from a longitudinal cohort study, and so the calibration is unaffected by surveillance underreporting and lags in reporting. The parameter values for underreporting and lag in reporting were chosen to explore the effect of these scenarios, and were not based on a particular reference. We added the following text to clarify this:**

“These scenarios were chosen to explore how under-reporting or reporting delays would affect the adaptive threshold strategies, and only relate to when and whether infections are counted towards the threshold for spraying. They do not affect the model calibration. Moreover, as the model was calibrated to serotype-specific estimates of infections based on serology from longitudinal cohort studies, the calibration is unaffected by surveillance underreporting and delays [49,51].” (lines 265-269)

2.4. Spraying parameters:

- They are not sufficiently clear before line 232. Their values are not clearly reported.

- I suggest to include the parameters and their values (and/or explored ranges) in a table (with some references?). These could be included in table 1.

- In line 233 the sentence “...plausible values...” is too vague.

**We added parameter values to Table 1, along with explored ranges. We also refer to this table earlier in the exposition (line 211).**

3. Results.

3.1. I would appreciate a comparison with the real reported number of infections, for example this could be added on table 2. Also, a real time series of dengue incidence in Iquitos would be valuable, which could be included in Figure 4.

**Because the ratio of infections to reported cases is known to fluctuate widely over time (from 1:1 to 40:1), the trend in reported cases may not accurately reflect the trend in the number of infections [1,2]. For this reason, we chose to calibrate our model to an estimate of the serotype-specific number of infections based on a longitudinal cohort study, which should provide a more accurate insight into the actual transmission dynamics. For the same reason, we refrained from comparing our timeseries to the one for reported cases. Instead, we compared our model output to the estimated number of infections in Supplementary Figure 1.**

3.2. Some axes names in Figure 3, Figure 4, and Figure 7 are missing.

**We have now added axes names to all figures.**

3.3. It might be a good idea to combine Figure 3 and Figure 7 to facilitate comparison between methods. In fact, in line 333, the authors ask to compare these two figures “...,(compare Figure 7 to Figure 3).”

**We added a summary plot to Figure 8 to aid comparison of ULV and TIRS, and changed the text to refer to this.**

3.4. The right and left columns of Figures 5 and 9 could be condensed into one.

**See point 3.5 below**

3.5. That is, the monthly and weekly results could be plotted in one graph, which would facilitate the comparison. Moreover, it may be good to produce only one figure with the information of figure 5 in one column and information of figure 9 in another column. The second row, the mosquito abundance, in Figure 5 is not discussed. In particular, I would appreciate a discussion on why as the threshold increases, the abundance of mosquitoes increases while the total number of infections “decreases” (I understood that it has a minimum for middle values). Moreover, with the TIRS (figure 9) this is different, both mosquitoes abundances and total infections increases as the threshold is higher. In addition, Figure 9 is practically not discussed in the results section (only one sentence).

**These are interesting suggestions to clarify the discussion of the fixed threshold results. We have now combined the ULV and TIRS results into one figure (Figure 6). As the results for monthly and weekly thresholds were similar, we just show the monthly results in Figure 6, in order to not obscure the relationships with threshold. We included a figure which compares monthly and weekly on the same plot in the supplementary material, however (Supplementary Figure 7).**

**We added the following text to discuss the point about mosquito abundance:**

“We also see a linear increase in mosquito abundance as we increase the threshold and reduce the amount of spraying, although the effect on average mosquito abundance is weak due to the short-lived effect of ULV.” (lines 328-330)

**Regarding the reason why an increasing threshold initially corresponds to a decreasing number of infections but increasing mosquito abundance, this was discussed in lines 327 to 339, and explored in Figure 6. We also added the following sentence to further clarify this:**

“Essentially, as the effect of ULV is short-lived, the timing of spraying is more important than the total amount of spraying over the 11-year period, meaning a lower threshold is not better if it means spraying occurs too early.” (lines 340-342`)

**Finally, to compare this observation with TIRS we added the following sentence:**

“Unlike for ULV, increasing the threshold for TIRS initiation tends to increase the number of cases. This is because timing is less important for TIRS, so spraying earlier in the season is not as big of a disadvantage as for ULV.” (lines 378-380)

4. Discussion. The discussion is broad, but it is a somewhat long, it could also be better organized. I suggest working on the writing of this section so that the main ideas are more straightforward.

**To make the Discussion easier to digest, we added subtitles and slightly rearranged the content, moving all discussion of limitations into one place.**

References

1.	Reiner RC, Stoddard ST, Forshey BM, King AA, Ellis AM, Lloyd AL, et al. Time-varying, serotype-specific force of infection of dengue virus. Proc Natl Acad Sci USA. 2014;111: E2694-2702. doi:10.1073/pnas.1314933111

2.	Morrison AC, Minnick SL, Rocha C, Forshey BM, Stoddard ST, Getis A, et al. Epidemiology of Dengue Virus in Iquitos, Peru 1999 to 2005: Interepidemic and Epidemic Patterns of Transmission. Tesh RB, editor. PLoS Neglected Tropical Diseases. 2010;4: e670. doi:10.1371/journal.pntd.0000670
